# Supplementary material for: Biosynthetic Gene Content of the ‘Perfume Lichens’ Evernia prunastri and Pseudevernia furfuracea
Source: Molecules. 2019 Jan 8;24(1):203. doi: 10.3390/molecules24010203 (PMC6337363; doi:10.3390/molecules24010203)

RAxML tree based on
• the protein sequence of the ketosynthase (KS) domain
• 550 entries (413 sequences from lichen-forming fungi, 131 characterized metabolites from MIBiG, 6 animal fatty acid synthases)

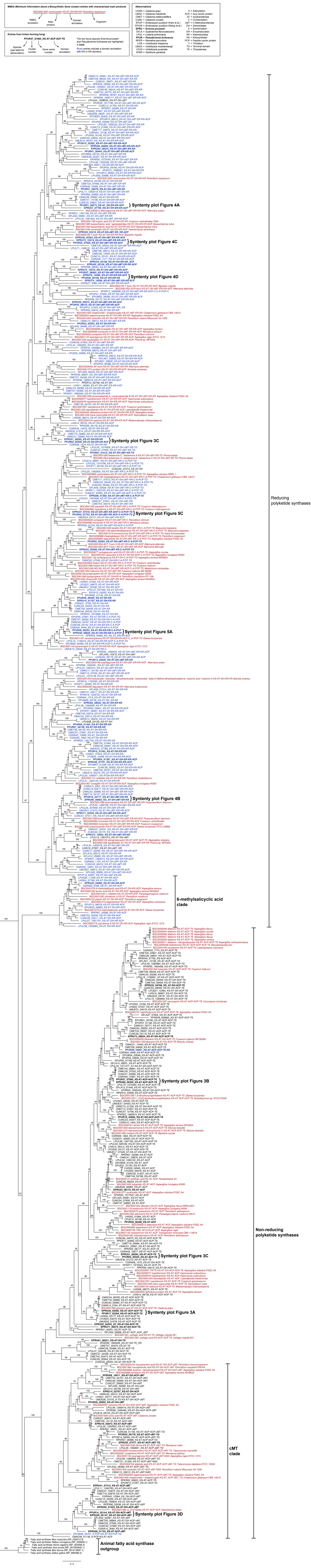

Supplement: Supplementary file 1 [file molecules-24-00203-s001.zip › Supplementary_Figure-S1.pdf]
